# The temperament of preterm infant in preschool age

**DOI:** 10.1186/1824-7288-37-4

**Published:** 2011-01-10

**Authors:** Giovanna Perricone, M Regina Morales

**Affiliations:** 1 Department of Psychology, Faculty of Educational Sciences, University of Palermo, Viale delle Scienze - Ed. 15, Palermo, 90128, Italy

## Abstract

**Background:**

The study deals with the characteristics of temperament of preterm infants during their preschool age in order to not only investigate likely "difficult or problematic profiles", guided by impairments driven by their preterm birth, but also to provide guidelines for the activation of interventions of prevention, functional to improve the quality of preterm infant's life.

**Methods:**

The study involved a group of 105 children where 50 preterm children at the average age of 5 years and 2 months, enrolled in preschools of Palermo. The research planned the child reference teachers to be administered a specific questionnaire, the QUIT, made up of 60 items investigating six specific typical dimensions of temperament (*Motor control activity *- related to the ability of practicing motor control activity; *Attention *- related to the ability of guiding and keeping the focus of attention on a certain stimulus; *Inhibition to novelty *- regarding with emotional reactivity in front of environmental stimuli; *Social orientation *- meant in terms of attention and interest towards social stimuli; *Positive and negative emotionality *- regarding the tendency to mainly express positive or negative emotions.

**Results:**

The results show in general how preschool-aged preterm infants, identified by such a study, compared with full-term children, are characterized by "normal" temperament based on a strong inclination and orientation in mainly expressing positive feelings. Yet, an impairment of the areas most relating to attention and motor control activity seems to emerge.

**Conclusions:**

The data suggest specific interventions for preterm infant development and their reference systems and, at the same time, can guide paediatrician and neonatologist dealing with preterm infants, in focalizing and monitoring, even since health status assessments, specific areas of development that, since preschool age, can highlight the presence of real forerunners of maladjustments and likely configurations of cognitive, emotional or behaviour disadaptive functioning.

## Background

Premature birth is an evolutional risk condition for children at their birth, for their survival and initial characteristics of their neonatal development (organic-functional immaturity due to gestation age - < 32 weeks, birth weight < 2000 gr., neurocognitive complications, heart and breath difficulties, muscle hypotony, poor reflexivity, etc.), and, mainly, for their entire evolutional course that it often seems to take shape even since his/her early childhood, in terms of behaviour, cognitive, social and motor control impairments [1-3]. Several studies, in fact, highlight how preterm infants, more frequently than full-term children do, show problematic evolutional outcomes that are often displayed at preschool and school age only. They are related with cognitive area (sensorial deficit, language impairments, learning difficulty, etc), emotional area (that is more specifically, impairments in regulation of emotions and impulses), and relational area (behaviour problems and of adjustment in social relationships with their peer group and adults) [4-11].

This study, just referring such a heuristic picture, following the Paediatric Psychology perspective [12-15], focuses its attention on some development areas of preschool-aged preterm infants. In particular, it studies the characteristics of their temperament [16-18], investigating likely "difficult or problematic profiles", guided by the impairments brought by their preterm birth and social and individual factors that interact with it, and providing guidelines to activate prevention interventions functional to the improvement of quality of life of preterm infants. They are typologies of information suggesting reference paediatricians the focuses of intervention and monitoring during health status assessments.

Therefore it can be mainly focalized specific areas of motor control development (movement coordination) or cognitive (attentive and regulative processes) or emotional (expression and modulation of emotions) that highlight, even since preschool age, the presence of real precursors of future maladjustments and likely disadaptive configurations of cognitive, emotional, maladjustment or behaviour functions [9-11].

In particular, the study model of reading the temperament is in terms of goodness of fit between individual and environment, meant as a variable moderating the reciprocal adjustment between child and environment [19-21] and referring to a series of cognitive, emotional and relational processes. *Motor control activity*, *Attention*, *Inhibition to novelty*, *Social orientation *and *Emotionality *are the model areas. Each of them is considered with a specific meaning.

The *Motor control activity *can be meant as the vigour of movement and modulation of motor control activity - the lack of such processes in preterm infants seems to guide some impairments on motor control development especially in relation to their capacity of modulating the "end" movement and visuospatial coordination [3].

The *Attention *area is meant in terms of orientation, regulation and attention persistency, that are all processes considered, in preterm infants, little relevant by literature, at the early phases of their life mainly. Preterm infants seem to be more distractible and less able in passing and linking an emotional internal phase to an external one, monitoring what happens or their own action [22], as well as they prove to be less able to keep attention focus on an object, or on what they are doing. Such unsteadiness does not seem to allow preterm infants to activate a series of mechanisms and other cognitive processes (collecting and selecting information, concentration, etc.) functional to a more rational adjustment to situations.

As for the *Inhibition to novelty*, the model refers to emotional reactivity introducing an adjustment to social context [2], under studies underlying that in preterm infants there is often a higher reactivity to external and internal stimuli than full-term children.

The *Social orientation *area is meant as emotional answers in front of unknown people and attention/interest towards social stimuli. Preterm infants seem to approach what is new with a greater self-confidence than full-term children do, and show a good involvement and openness towards interpersonal relationships [17].

Finally, in relation with *Emotionality *area, the model focalizes the predominance of negative and positive emotions referring to preterm infants' higher predisposition and orientation toward expressing positive emotions [17].

Furthermore, the model states that such areas give life to profiles that, more or less characterized by the impairment of certain processes [23-26,17], evolve during their development, up to reduce likely differences between preterm and full-term children. Therefore, from a social view of preterm infants as "difficult-temperament" children [19], a view based on "easiness", sociability and patience was reached [27-29,18].

Considering the cultural contextualization of such studies (Australia, England, U.S.A., etc.), this study is aimed to verify the likely overlapping of data and then, their cross-cultural validity.

## Methods

### Objectives and hypothesis

In light of the last considerations and in function of the above described model, the study has the following goals:

- Investigating the characteristics of temperament areas of preschool-aged preterm infants

- Investigating the temperament profile (emotive, calm, normal, difficult) of preschool-aged preterm infants

Considering such aims, research hypothesis are to be found in:

- Verifying the existence of statistically significant differences between preterm infants and full-term children, with regard to the different areas defining temperament (Motor control activity, Attention, Inhibition to novelty, Social orientation and Emotionality)

- Verifying the existence of statistically significant differences temperament among temperament profiles (emotive, calm, normal, difficult) of preterm infants and preschool-aged full-term children.

### Participants

The research group (Table 1), was made up by 105 children at the average age of 5 years and 2 months. Almost every child, whose characteristics were studied, had siblings (usually 2) and belonged to families of middle social class (average one-incoming families where parents had an average education level of secondary school). Children, were preschool aged, and they are enrolled in schools of Palermo and province.

**Table 1:** Sample characterists

Characteristics of children born preterm (= 50)				Characteristics of children born full - term (= 55)		
***Variable***	***mean***	***ds***	***range***	***mean***	***ds***	***range***

Child age (months)	62	4	57-67	64	2.5	61-66

Birth Gestational Age	29	2	27-31	40	2.5	39-41

Birth Weight (g)	1800	350	1450-2400	2600	800	2300-3400

Days of Hospitalization	15	8	8-23	2	1.5	2-3

**Family background** **of children born preterm**				**Family background** **of children born full term**		

***Variable***	***mean***	***ds***	***range***	***mean***	***ds***	***range***

Age of Parents (years)	30,6	6	24-37	32,6	5	28-38

Education of parents (years)	13	8	8-23	12	8	8-22

Number children	2	1.5	1-3	2	1.5	1-4

The involved children were divided into two groups: an experimental group, so defined because of the presence of the variable "preterm birth", and a control group. The experimental group was made up by 50 preterm premature children, with low birth weight (mean gestational age = 29 weeks, ds = 2; mean birth weight = 1800 gr., ds = 350 gr.), selected in function of the following criteria: gestational age < 32 weeks, birthweight 1500 to 2500 gr. and lack of neurologic pathology, sensorial deficit and genetic pathology or malformative syndrome. The control group was made up by 55 full-term children (mean gestational age = 40 weeks with no pre- and perinatal complications), with the same anamnestic and sociocultural characteristics of the experimental group (cfr. Table 1). The selection criteria of the control group were: about 40^th ^post conceptional week at birth (range = 39-41 gestational weeks), birthweight >2500 gr., lack of pre- and perinatal complications, and lack of neurologic pathology, sensorial deficit and genetic pathology or malformative syndrome.

Every child was involved in the research after getting a declaration of approval of their parents, who were informed about aims and procedures of research path to which the study is referred, they were requested to sign a data informative, under art.13 of D.LGS. 196/2003 granting people protection and other subjects in relation to personal data treatment.

### Procedures and instruments

A questionnaire was used to observe the behaviour of children aged 3 to 6 years, belonging to the battery of Temperament Italian Questionnaires (QUIT), validated on Italian sample [21].

Such a questionnaire, which can be filled in by parents (even with a low, medium-low level of education), educators and teachers, or however, anyone who, taking care children, spends his/her time with them every day, was administered to infancy school teachers that child had been attended for 3 years.

The questionnaire is structured in 60 items describing child behaviour in three different contexts (child with the others; child on his play time; child facing of novelty or while s/he is performing an activity or a task), and the answers teachers can give are "almost never" [1] to "almost always" [6], under the Likert scale. The items refer to the six areas and dimensions previously described, each explored through 10 items (*Motor Control Activity *items n.11, 13, 15, 16, 34, 36-40, 45, 59; *Attention *items 41-44, 47, 54, 57, 58; *Inhibition to novelty *items 26, 29, 32, 35, 48-53, 56, 60; *Social Orientation *items 1-10; *Positive Emotionality *items 12, 17, 21, 23, 25, 27, 28, 31, 46 and *Negative Emotionality *items 14, 18-20, 22, 24, 30, 33).

More specifically, three areas are related to child's adjustment to environment in general: the *Motor activity *area, regarding the ability of performing motor activity, the *Attention *area regarding the ability of orienting and keeping the attention focus on a certain stimulus, and the *Inhibition to novelty *area related to the emotional reactivity to environmental stimuli.

The other three areas regard child's adjustment to social world and, in particular *Social orientation *area is meant in terms of attention and interest in social stimuli, *Positive emotionality *and *Negative Emotionality *areas refer to the prevalence of positive or negative emotions. The last two scales of QUIT (*Positive emotionality *and *Negative emotionality*) allow to clearly assess the emotional component of temperament ("quality of mood", 19), and highlight 4 temperament profiles, most consistent with Italian cultural context:

1. *Emotional temperament*, typical of individuals with high emotional reactivity, who easily cry and laugh. They correspond to the definition of < < lively> >, nice and emotional child. Such a profile plan that the score obtained by the subject is higher than the mean value of the normative sample in the scale of both Positive emotionality (E+) and Negative emotionality (E-).

2. *Calm temperament*, typical of individuals showing a low emotional reactivity. They smile instead of laughing and get angry, cry or get frightened rarely. These children get a score lower than the mean value of the normative sample in the scale of both Positive emotionality (E+) and Negative emotionality (E-).

3. *Normal temperament *regarding those individuals showing a prevalence of positive emotions since the first months of their life. These children, having high positive reactivity and low negative reactivity, obtain a score higher than the mean value of the normative sample in the scale of Positive emotionality (E+) and a lower score in the scale of Negative emotionality (E-).

4. *Difficult temperament*, describes those individuals where negative emotions prevail against positive ones. They are children whose interactions with environment are often difficult and the child-environment adjustment is extremely problematic. They obtain a score in the scale of Negative emotionality (E-) higher than the mean value of the normative sample, while a lower score in the scale of Positive emotionality (E+).

In relation to the psychometric characteristics of the questionnaire, there is the need to specify that it was validated on a great number of subjects (n = 1533) by means of a repeated administration to both parents and child's reference teachers, performed in several Italian cities.

More specifically, as for reliability and internal validity of the questionnaire, the internal consistence of the dimensions was calculated through Cronbach's alpha, which highlighted an acceptable cohesion among QUIT dimensions (α > .60 in every dimensions) [21]. Furthermore, correlational analysis was performed among the scales of the questionnaires filled in by fathers, mothers and teachers of children, by means of Pearson's correlation coefficient that highlighted the capacity of questionnaires to measure the objective aspects of temperament (R > .52 e p < .01) [21].

### Data treatment and analysis

Data codified under the procedures set by the reference test guide, were analyzed by means of the statistical programme for Social Sciences - SPSS (16th version for Windows).

More specifically, in relation to the survey on likely differences between preterm and full-term children, within the different areas defining their temperament (QUIT), an analysis of one way variance (ANOVA) for continuous variables (scores related to different scales) was performed, and through Kolmogorov Smirnov's test [30], it allowed to compare the sample means of preterm infant group - experimental group/preterm birth independent variable - to those of children born after a normal gestation - control group/on time birth independent variable - within the different scales of QUIT. As for the test of significativity, a value of p = 0,05 was used.

## Results and Discussion

The results of analysis on the differences between preterm and full-term children in the different areas of temperament, highlighted the existence of specific differences (Table 2).

**Table 2:** Temperament dimensions in preterm and full-term children

*Scales*	*Mean* *preterm children*	*ds*	*Mean* *full-term children*	*ds*	*F*	***Sign***.
Social orientation	4,31	0,94	4,42	0,74	,496	,483

Inhibition to novelty	3,04	0,74	3,13	0,97	,253	,616

Motor control activity	3,07	1,11	3,27	1,27	,997	,320

**Positive emotionality **(E+)	4,3	0,5	3,91	0,82	7,589	,007

Negative emotionality (E-)	2,8	0,66	3,02	0,87	2,049	,155

Attention	3,5	0,82	3,6	0,87	,456	,501

**Figure 1:**
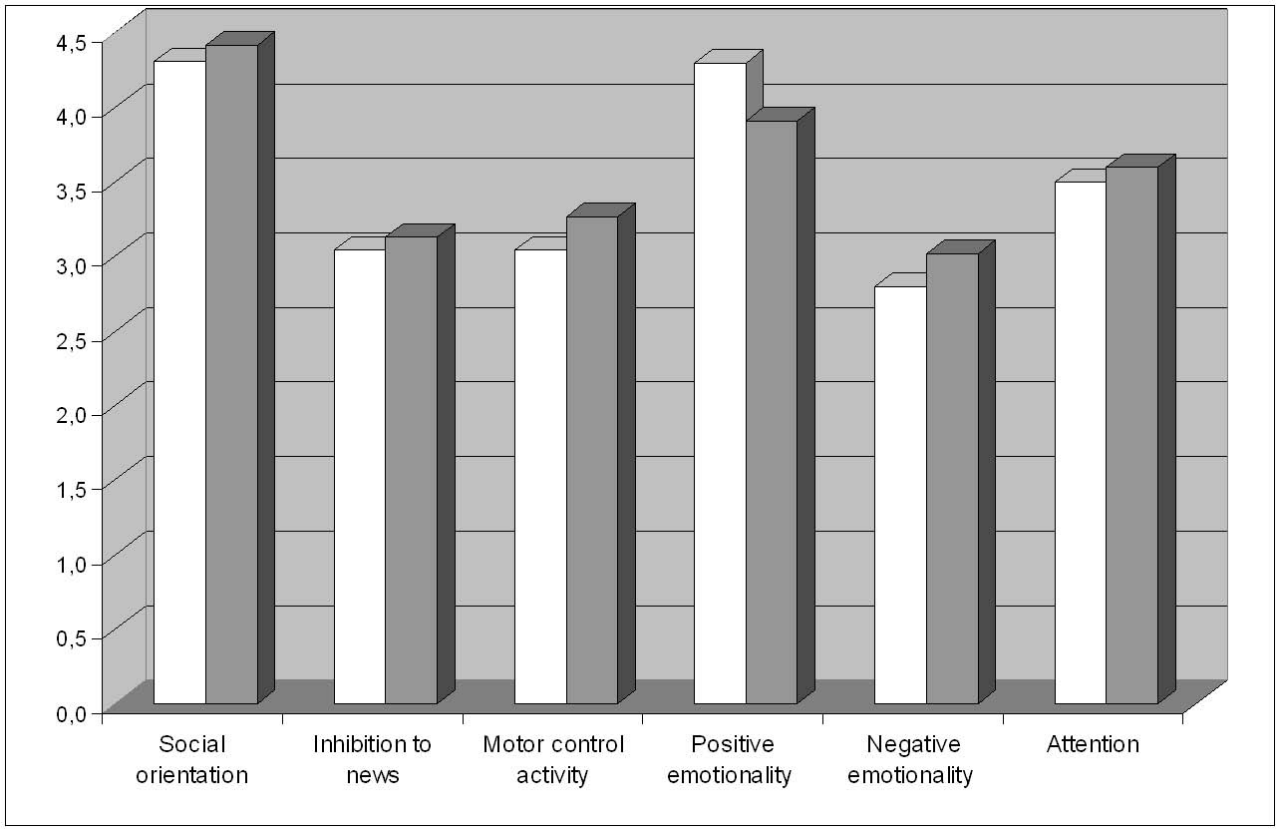
**Dimension of temperament in preterm and full-term children**. - White: preterm children. - Gray: full-term children

Statistically speaking, preterm infants seem to significantly differ from full-term children only in the scale related to Positive emotionality (p < .05) where they obtained a higher score than full-term children had, due to their higher predisposition toward expressing positive feelings and experiences. With regards to the other scales of QUIT (*Social orientation*, *Inhibition to novelty*, *Motor control activity*, *Negative emotionality*, *Attention*) preterm infants obtained lower scores compared to full-term children, even though such a difference does not have a statistic significance (p > .05) (see Table 2; Figure 1).

Moreover, as for the possibility to assess the different temperament profiles of preterm and full-term children, a descriptive comparison between mean scores and standard deviations of preterm and full-term children and the mean score and standard deviations of the normative test sample was performed in the Negative and Positive Emotionality scales (Table 3).

**Table 3:** Preterm and full-term children temperament profile

*QUIT temperament scales*	*mean score normative sample*	*ds*	*mean* *preterm children*	*ds*	*mean* *full-term children*	*ds*
Social orientation	4,06	0,43	4,31	0,94	4,42	0,74

Inhibition to novelty	3,25	0,69	3,04	0,74	3,13	0,97

Motor control activity	3,66	0,46	3,07	1,11	3,27	1,27

Attention	3,82	0,72	3,5	0,82	3,6	0,87

**Positive emotionality**	4,03	0,52	4,3 +	0,5	3.91 -	0,82

**Negative emotionality**	3,37	0,58	2,8 -	0,66	3,02 -	0,87

**Temperament profile typology**			***Normal***		***Calm***	

In relation to the specific temperament profile, preterm infants seem to be described with a normal temperament, showing high positive reactivity and low negative reactivity (mean score in E+ is higher than mean score in E-); on the other hand, full-term children, getting a low score in both E+ and E-, show less emotional reactivity and hence, highlight a calm temperament.

The results of such a study show how preschool-aged preterm infants, compared to full-term children are characterized by a temperament profile that, within Italian culture is defined in terms of "normality" and, hence, based upon a strong predisposition and orientation toward mainly expressing positive emotions. However, although a statistical significativity was not reached, preterm infants got slight lower scores in all other dimensions of temperament but in the positive emotional scale (see Table 2). So, preschool aged preterm infants involved in the research, seem to be also characterized by low levels of motor control activity, attention and negative emotive reactivity, by a predisposition toward motor and attention irregularity, and difficulty in recognizing and expressing negative emotions. Finally, preterm infants have a low score of social orientation meant as the relational curiosity functional to promote certain self-regulated answers of adjustment to external reality.

## Conclusions

Such a study, though considering the small number of the sample, highlights a temperament profile of preschool-aged preterm infants whose specificity, compared to full-term children involved in the research path, is to be found in strong predisposition and orientation toward expressing positive emotions rather than negative ones, and a high trend toward searching the other and, hence, being sociable.

Moreover, it was detected the presence of a sort of "slowness" in preterm infants involved in the research, that, even though does not reach a statistical significativity, is mainly related to both motor and attention fields. So, it is to be highlighted a likely difficulty of motor control development in preschool-aged preterm infants, showing low levels of motor control activity, a less motor reactivity and difficulty of coordination and minor endurance of it [3]. The presence of such elements was detected by the literature in preterm infants compared to full-term children [17,18].

Another aspect to be considered is related to preschool-aged preterm infants' attention. In accordance with several studies [22], a minor trend toward guiding and regulating their own attention, keeping focalization on an object, and a minor capability of moving their attention from a stimulus to another one, were highlighted. They all are processes whose impairment may be a likely risk factor for a rational adjustment to situations, and drive to difficulties in school learning.

In light of these last considerations, such a study is aimed to open new and future hypothesis on the existence of likely correlations between such temperament aspects and specific cognitive, emotive and relational functions of preterm infants. On the other side, it can guide paediatrician and neonatologist or, more generally, preterm infants cure system, in more focalizing and monitoring, within follow-up paths and related health status assessments, those cognitive, relational or motor control areas, correlated to temperament and that research data highlight in some way impaired. They are, in fact, extremely important processes for child's development [31-33], so that even the little impairment of them, seems to come with specific forms of evolutional maladjustments such as disorders and/or deficit of attention [3,22], hyperactivity or disorders in emotive self-regulation [2], learning [34-37] and language disorders [38] that tend to guide the preterm infants' life path in terms of "atypical" path [39].

A further element that the study wants to highlight is related to cross-cultural perspective of data, that is, research preterm infants seem to be characterized by a temperament profile that, in its general configuration, can overlap to that highlighted by other studies performed on children coming from cultures deeply different from the considered one (United States, England, Australia, etc.) [33,17,2]. More specifically, while in Italian culture, the profile of temperament dimensions is defined in terms of "normality" and hence, adaptive to the reference socio-cultural context, in other cultures it is read using different parameters and models, and therefore, interpreted in terms of "difficult" functioning, that is not functional to adjustment to reality [40-43]. Such a consideration, even though it does not disregard the importance of cultural attributions that guide specific temperament profiles, leads to hypothesize and question about a likely presence of a sort of "temperament syndrome" [40] of preterm infants, meant as the existence of a configuration of temperament of preterm infants, made up by a strong constitutional base with a cross-cultural validity. Moreover, to support such hypothesis, it could be also considered that, while other studies have detected this temperament configuration of 0-to-2-year-old preterm infants, preterm children involved in such a research are all 5 years old. So, it has been supposed the permanence of such a syndrome during the development, that would lead to define preterm infants compared to full-term children, even school-aged, in terms of emotive, sociable and patient children yet less directive, attentive and reactive to frustrations.

## Ethical approval

Not required. This is a retrospective study. Informed consent for developmental evaluation was given by parents of each child, under art.13 of D.LGS. 196/2003 granting people protection and other subjects in relation to personal data treatment.

The study was realized with the contribution of University of Studies of Palermo

## Competing interests

The author declares that they have no competing interests.

## Authors' contributions

The authors contributed equally to this work. The authors read and approved the final manuscript.
